# Prevalence of complicated carotid atherosclerotic plaques ispilateral to ischemic cryptogenic stroke using high-resolution mri

**DOI:** 10.1186/1532-429X-13-S1-P379

**Published:** 2011-02-02

**Authors:** Tobias Saam, Tobias Freilinger, Andreas Schindler, Jochen Grimm, Caroline Schmidt, Fabian Bamberg, Martin Dichgans, Chun Yuan, Maximilian F Reiser, Konstantin Nikolaou

**Affiliations:** 1 University of Munich, Munich, Germany; 2 University of Washington, Seattle, WA, USA

## Introduction

Although distinct pathogenetic mechanisms for ischemic stroke have long been recognized, a definite or even probable etiology can not be established in about one third of all patients (“cryptogenic strokes”). Recent studies have shown that high-resolution carotid MRI is able to identify complicated American Heart Association lesion type VI (AHA-LT6) with hemorrhage, thrombus or rupture of the fibrous cap with good correlation to histopathology.

## Purpose

The purpose of our study was to evaluate the prevalence of AHA-LT6 in carotid arteries of subjects with cryptogenic stroke.

## Methods

30 consecutive patients (24 men, mean age 69.9 ± 11.9 years) with cryptogenic stroke *and* intimal thickening by duplex sonography were recruited from our stroke unit. All patients underwent extensive clinical workup (lab, brain MRI, duplex sonography, 24-hour ECG, transesophageal echocardiography) to exclude carotid stenosis ≥ 50%, cardiac embolism, small vessel disease and other causes of stroke. All subjects received a high-resolution carotid black-blood MRI at 3.0-Tesla with fat-saturated pre- and post-contrast T1w-, PDw-, T2w- and TOF images using surface coils and Parallel Imaging techniques (PAT factor=2). Prevalence of AHA-LT6 was determined in both carotid arteries based on previously published MRI criteria by two experienced reviewers who were blinded to the clinical information.

## Results

**Figure 1:**
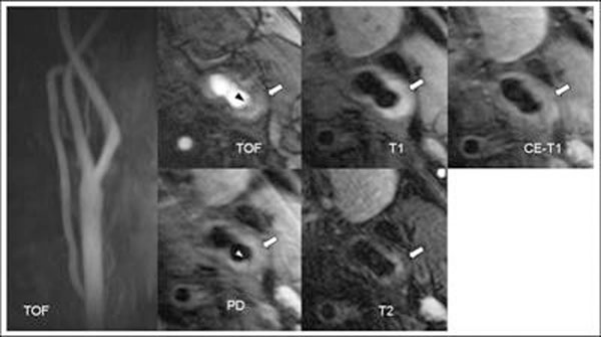
Images of a non-obstructive complicated AHA lesion type VI of a 66-year old male patient with a ,,cryptogenic“stroke ipsilateral to the lesion. The triangles on the TOF and PDw Images point to the site of the plaque rupture. The arrows point to a large lipid/necrotic core with type I Hemorrhage which does not cause substantial luminal narrowing (see TOF images on the left).

AHA-LT6 with hemorrhage, rupture of the fibrous cap and / or thrombus were found in 14 out of 30 arteries (46.7%) ipsilateral and in 1 out of 30 arteries (3.3%) contralateral to the ischemic, “cryptogenic” stroke (*P<0.001*). Of the 15 plaques classified as AHA-LT6, 13 had plaque hemorrhage, 2 had mural thrombi and 8 a rupture of the fibrous cap. Figure 1.

## Conclusions

Complicated non-stenotic carotid atherosclerotic lesions were found significantly more often ipsilateral than contralateral to ischemic “cryptogenic” stroke.

